# Interaction between Macrophages and Human Mesenchymal Stromal Cells Derived from Bone Marrow and Wharton’s Jelly—A Comparative Study

**DOI:** 10.3390/pharmaceutics13111822

**Published:** 2021-11-01

**Authors:** Marta Dymowska, Aleksandra Aksamit, Katarzyna Zielniok, Monika Kniotek, Beata Kaleta, Aleksander Roszczyk, Michal Zych, Filip Dabrowski, Leszek Paczek, Anna Burdzinska

**Affiliations:** 1 Department of Immunology, Transplantology and Internal Diseases, Medical University of Warsaw, Nowogrodzka 59, 02-006 Warsaw, Poland; marta.dymowska@wum.edu.pl (M.D.); aleksandra.aksamit31@gmail.com (A.A.); leszek.paczek@wum.edu.pl (L.P.); 2 Laboratory of Cell Research and Application, Medical University of Warsaw, Banacha 1B, 02-097 Warsaw, Poland; 3 Department of Clinical Immunology, Medical University of Warsaw, Nowogrodzka 59, 02-006 Warsaw, Poland; monika.kniotek@wum.edu.pl (M.K.); beata.kaleta@wum.edu.pl (B.K.); aleksander.roszczyk@wum.edu.pl (A.R.); michal.zych@wum.edu.pl (M.Z.); 4 Department of Gynecology and Obstetrics, Medical University of Silesia, Medykow 14, 40-752 Katowice, Poland; filip.dabrowski@sum.edu.pl; 5 Department of Bioinformatics, Institute of Biochemistry and Biophysics, Polish Academy of Sciences, Pawinskiego 5A, 02-106 Warsaw, Poland

**Keywords:** mesenchymal stromal cell, MSCs, bone marrow, Wharton’s jelly, macrophages, migration, chemotaxis

## Abstract

Despite intensive clinical research on the use of mesenchymal stromal cells (MSCs), further basic research in this field is still required. Herein, we compared human bone marrow MSCs (BM-MSCs, *n* = 6) and Wharton’s jelly MSCs (WJ-MSCs, *n* = 6) in their ability to interact with human primary macrophages. Evaluation of secretory potential revealed that under pro-inflammatory stimulation, WJ-MSCs secreted significantly more IL-6 than BM-MSCs (2-fold). This difference did not translate into the effect of MSCs on macrophages: both types of MSCs significantly directed M1-like macrophages toward the M2 phenotype (based on CD206 expression) to a similar extent. This observation was consistent both in flow cytometry analysis and immunocytochemical assessment. The effect of MSCs on macrophages was sustained when IL-6 signaling was blocked with Tocilizumab. Macrophages, regardless of polarization status, enhanced chemotaxis of both BM-MSCs and WJ-MSCs (*p* < 0.01; trans-well assay), with WJ-MSCs being significantly more responsive to M1-derived chemotactic signals than BM-MSCs. Furthermore, WJ-MSCs increased their motility (scratch assay) when exposed to macrophage-conditioned medium while BM-MSCs did not. These results indicate that although both BM-MSCs and WJ-MSCs have the ability to reciprocally interact with macrophages, the source of MSCs could slightly but significantly modify the response under clinical settings.

## 1. Introduction

Mesenchymal stromal cells (MSCs) have been shown to be promising immunomodulatory agents. It was demonstrated that MSCs possess anti-inflammatory, tolerogenic, and pro-healing activities [1]. This population primarily resides in the bone marrow (BM), where they co-create an environment for the proper development of hematopoietic cells. However, cells with MSCs characteristics can be relatively easily isolated from different adult and perinatal tissues and expanded in vitro. This provides the possibility of MSC transplantation to modulate the immune system response in pathological conditions. The door to these possibilities is opening wider and wider. In 2017, MSCs were first approved as a drug in Europe (for patients with complex anal fistulas in Crohn disease [2]), and several other MSC preparations were approved in other parts of the world, such as South Korea, Japan, Canada, or India (presented in a review [3]). Although views on the clinical potential of MSCs have been evolving significantly over the past 20 years, there is now a relative consensus that the mechanism of action of MSCs, although not fully understood, is predominantly based on their effects on other cells, mainly cells of the immune system. Therefore, MSCs can exert an immunomodulatory effect as well as promote tissue healing/regeneration. Nevertheless, there is also consensus that many issues related to the clinical use of MSCs remain to be determined. It is known that MSCs derived from different sources, although sharing the same basic characteristics, differ in detail. For example, Wharton’s jelly-derived MSCs (WJ-MSCs) have been shown to secrete significantly less VEGF than adipose tissue-derived MSCs (AT-MSCs) [4] and significantly more TGF-β than AT-MSCs and BM-MSCs [4,5]. This may mean that certain types of MSCs will be more suited to certain clinical applications than others.

Among immunomodulatory activities of MSCs, the best documented is their inhibitory effect on lymphocyte activation and proliferation [6,7,8]. However, another potentially very powerful activity is the impact of MSCs on macrophages. Macrophages constitute a multifunctional group of cells essential in the processes of inflammation and regeneration. Depending on the signals coming from the local environment, they can acquire different phenotypes, which are simplistically classified as pro-inflammatory M1 phenotype or anti-inflammatory M2 phenotype [9]. Moreover, macrophages, due to their high plasticity, are able to transdifferentiate from one form to another, as well as remain in various intermediate states. It has been shown that MSCs can drive macrophage differentiation toward M2-like phenotype, and this mechanism was demonstrated both in vitro [10] and in vivo [11]. M2 macrophages suppress the pro-inflammatory activity of other immune cells and promote healing and regeneration. Therefore, M2 macrophages, along with regulatory T cells, are very desirable populations in disorders associated with excessive inflammation, including autoimmune diseases. Although it was previously shown by independent groups of researchers that both hBM-MSCs and hWJ-MSCs can polarize macrophages into an anti-inflammatory phenotype [10,12], a direct comparison of hBM-MSCs and hWJ-MSCs in terms of their influence on macrophages has not been published so far. On the other hand, some previous publications suggest that MSCs isolated from bone marrow and umbilical cord (Wharton’s jelly) may differ in their immunomodulatory potential as they display marked differences in the transcriptome [13] and differences in the expression/secretion of some factors known to be important for immunomodulation [5]. Therefore, the main objective of this study was to directly compare the effects of human BM-MSCs and WJ-MSCs on the phenotype of macrophages. We hypothesized that the two cell types might differ in their effects on the macrophage phenotype, which would indicate that one type of MSC may have greater clinical potential than the other.

Considering the effects of MSCs on macrophages, it is important to remember that macrophages are active participants in shaping the local tissue environment. Therefore, the interactions between MSCs and macrophages should be considered as bidirectional [14]. One of the key actions of macrophages (especially when activated) is the secretion of chemokines, which affect the composition of cells appearing nearby and may influence the local immune status. In terms of the potential clinical application of MSCs, this feature seems to be particularly important. It is clear that crucial for the therapeutic effect of MSCs is their delivery at the right site and in the right concentration. On the other hand, it has been shown that mobilization of transplanted MSCs to their target site, although it takes place, is often not sufficiently effective [15,16]. It is known that different subsets of macrophages release various chemokines attracting different cell types. MSCs were shown to express several chemokines receptors, including those that can interact with macrophage-derived chemokines, i.e., CCR3 interacting with CCL5 (secreted by M1 macrophages) and CCR4 interacting with CCL17/CCL22 (secreted by M2 macrophages) [17,18]. Therefore, the second aim of the present study was to evaluate the extent to which MSCs are attracted by different macrophage subsets and to compare the migratory activity of BM-MSCs and WJ-MSCs in the presence of macrophage-derived factors.

## 2. Materials and Methods

### 2.1. MSCs Isolation and Culture Methods

#### 2.1.1. Human Bone Marrow-Derived Mesenchymal Stromal Cells (hBM-MSCs)

Bone marrow samples were collected from 6 patients during standard orthopedic surgeries after obtaining their informed consent (4 male donors; age: 68, 39, 39, and 70 years old and 2 female donors, age 49 and 70 years old). The procedure was conducted with the permission of the local bioethics committee (approval number: KB/115/2018). BM-MSCs were isolated in accordance with the protocol described previously in detail [16]. Cells were plated on cell culture dishes in standard growth medium (GM) composed of DMEM low glucose supplemented with 10% fetal bovine serum, 1.0% penicillin-streptomycin, and 0.5% amphotericin B (all reagents from Biowest, Riverside, MO, USA). They were cultured in the standard conditions: 37 °C, 5% CO_2_, 95% humidity for four days with fresh medium replenishment after 48 h. The growth medium was subsequently changed, and non-adherent cells were rinsed away. The remaining adherent fibroblast-like cells were grown until reaching the subconfluency and were considered as mesenchymal stromal cells.

#### 2.1.2. Human Wharton’s Jelly-Derived Mesenchymal Stromal Cells (hWJ-MSCs)

Approximately 5 cm umbilical cord (UC) fragments were obtained from 6 patients after the planned delivery by cesarean section, with the approval of the Local Bioethics Committee (approval number: KB/32/2018). The sex of newborns (from which the UC originated) was not recorded. Samples were washed from blood and debris, placed in the sterile phosphate-buffered saline (PBS) with antibiotic solution (1.0% Penicillin-Streptomycin), and delivered within an hour to the cell culture laboratory. The connective tissue of the obtained umbilical cords (Wharton’s jelly) was divided with a scalpel into fragments (about 3 mm^3^) and transferred into cell culture dishes (ø 100 mm) with GM. The explants were maintained in standard conditions (37 °C, 5% CO_2_, 95% humidity) with fresh medium replenishment every 48–72 h, until migrating, adherent fibroblast-like cells were observed. Afterward, tissue fragments were removed from the culture dishes, and the GM was changed, following gentle PBS washing. The remaining Wharton’s jelly mesenchymal stromal cells were cultured until they reached the subconfluence.

Both cell types (hBM-MSCs and hWJ-MSCs) were identified after the 4th passage by multilineage differentiation and flow cytometry analysis. Cells on the 3rd–4th passage were frozen in liquid nitrogen for further use. In order to minimize the impact of in vitro senescence on the comparative study result, care was undertaken that the average total culture time of MSCs from various sources did not differ significantly. MSCs, regardless of their origin, were used for all experiments on the 4th–6th passage. MSCs from different donors were always cultured and tested separately.

### 2.2. MSCs Identification

According to current guidelines, isolated cells were identified by the expression of surface markers and the ability to three-lineage differentiation. The cell-surface antigen profile (CD44, CD73, CD90, CD105, CD11b, CD19, CD34, CD45, and HLA-DR) was evaluated on a BD FACS Canto II with BD FACS Diva Software (Becton Dickinson, Sparks, NV, USA) using the BD Stemflow™ hMSC Analysis Kit (BD Biosciences, San Jose, CA, USA). To assess differentiation, cells were cultured in adipogenic, osteogenic, and chondrogenic conditions as described in detail previously [19] and stained with Oil Red O, Alizarin red, and toluidin blue, respectively (all reagents for staining from Sigma-Aldrich, Saint Louis, MO, USA).

### 2.3. Human Peripheral Blood Mononuclear Cells (PBMCs) Isolation and Mixed Lymphocytes Reaction (MLR)

Peripheral blood mononuclear cells were isolated from the buffy coats of healthy anonymous donors that were obtained from the Regional Blood Donation and Blood Treatment Center. Buffy coats were diluted 1:2 in PBS, and after density gradient centrifugation with Histopaque-1077 (Sigma-Aldrich, Saint Louis, MO, USA) (20 min, RT), the interphase containing PBMCs was collected. PBMCs were washed subsequently by centrifugation in PBS in order to rinse cells and reduce platelets number (3 × 1300 rpm for 8 min and 1 × 1000 rpm for 10 min). The day before MLR assay, BM-MSCs and WJ-MSCs (cells from 5 donors of each cell type were used for this assay) were seeded in 96-well flat-bottom plates (Greiner Bio-One GmbH, Kremsmünster, Austria) in a density of 0.8 × 10^4^/well. For each set of experiments, PBMCs were isolated from 2 unrelated donors and suspended in RPMI1640 (Biowest, Riverside, MO, USA) supplemented with 2 mM L-glutamine, 0.1 mg/mL gentamycin (KRKA), β-mercaptoethanol, 0.2% HEPES (all from Sigma-Aldrich, Saint Louis, MO, USA) and 10% fetal bovine serum (FBS, Gibco, Thermo Fisher Scientific, Waltham, MA, USA). Half of the isolated PBMCs from each donor were inactivated by γ-irradiation for 90 min. A total of 2 × 10^5^ PBMCs (1 × 10^5^ cells/well from a first donor (A) and 1 × 10^5^ cells/well from the second donor (B)) were seeded to the wells with attached BM-MSCs and WJ-MSCs in the following combinations: AAir, BBir, ABir, BAir. PBMC cultures without MSCs were used as controls. After 5 days of culture in standard conditions, the cells were pulsed with 1 μCi/well of 3H-thymidine (113 Ci/nmol, NEN) for the last 18 h of the incubation and harvested with an automated cell harvester (Skatron, Lier, Norway). The amount of 3H-thymidine incorporated into the cells was measured using a Wallac Microbeta scintillation counter (Wallac Oy, Turku, Finland), giving the level of radioactivity as ‘Corrected Counts per Minute’ (CCPM). Experiments were performed in triplicates. In this study, PBMCs from 10 donors and MSCs from 5 donors (of each cell type) were used for MLR assay.

### 2.4. Monocytes Isolation and Stimulation of Macrophages

Monocytes were obtained from PBMCs by the adhesion method, as we previously described [19]. PBMCs were suspended in RPMI1640 (Biowest, Riverside, MO, USA) without serum and seeded at a concentration of 1 × 10^6^ PBMC/cm^2^ culture dish. After 2 h of incubation in standard conditions (37 °C, 5% CO_2_, 95% humidity), cells were rinsed with PBS to remove unattached cells. The remaining adherent cells, which were significantly enriched in CD14+ cells, were cultivated in medium consisting of RPMI1640 supplemented with 10% human serum (HuS, cat. No. H3667, Sigma-Aldrich, Saint Louis, MO, USA) and antibiotics (1% penicillin-streptomycin) in standard conditions. They were left for 3 days to acquire the macrophage phenotype. After 3 days of culture, the medium was changed, and the monocytes/macrophages were either remained untreated (M0) or were stimulated with: (1). IFNγ (cat. No. RIFNΓ50, from Thermo Fisher Scientific, Waltham, MA, USA) in concentration 10 ng/mL; M_IFNγ_, (2). IFNγ and lipopolysaccharide (Sigma-Aldrich, Saint Louis, MO, USA), both in concentration 10 ng/mL; M1 (3). Interleukin (IL)-4 and IL-10 (cat. No 11340043 and 11340103, from ImmunoTools GmbH, Friesoythe; Germany), both in concentration 10 ng/mL; M2. The process of stimulation lasted 4 days. The fresh portion of cytokines was added every other day.

### 2.5. Direct Co-Culture of Human MSCs and M_IFNγ_ Macrophages

Mesenchymal stem cells were seeded to the M_IFNγ_ macrophages at a ratio of 1:10 on the 7th day of culture. In each experimental set, macrophages from one patient were co-cultured with both BM- and WJ-MSCs. MSCs were stimulated with IFN-γ (25 ng/mL) for 24 h prior to the co-culture. It is known that the treatment of MSCs with pro-inflammatory agents (e.g., IFNγ) enhances their immunomodulatory activity. Stimulation was performed prior to introducing the MSC into co-culture with macrophages to maintain the reproducible strength of the stimulus across the various experiments of this study. The co-culture was carried out for the next 3 days in standard macrophage medium (RPMI1640 with 10% HuS) without the addition of cytokines. Each time, macrophages from the same donor cultured in the same conditions but without the addition of MSCs served as control. For some experiments, directly before seeding MSCs to macrophages, mesenchymal stromal cells were stained with red fluorochrome DilC18(5)-DS (1,1′-Dioctadecyl-3,3,3′,3′-tetramethylindodicarbocyanine-5,5′- disulfonic acid, DID) (AAT Bioquest, Sunnyvale, CA, USA). Next, the effects of BM- and WJ-MSCs on the expression of macrophage cell-surface antigens were assessed using flow cytometry and immunocytochemistry methods.

### 2.6. Flow Cytometry Analysis of Macrophage Immunophenotype

For flow cytometry, macrophages were cultured on ø 60 mm Primaria™ (Corning, Tewksbury, MA, USA) cell culture dishes. At the end of the experiment, cells were gently harvested with a cell scraper in cold FACS staining buffer (PBS with 2% FBS and 0.1% sodium azide), counted, and centrifuged (1300 rpm, 7 min, RT). Next, they were resuspended in the solutions of antibody in staining buffer and incubated for 20 min on ice in the dark. After washing, samples were acquired with Becton Dickinson FACS Canto II equipped with a 488 nm laser, a 633 nm laser, and a 405 nm laser. The data were analyzed with the FACS Diva 6.1.3. software. Macrophage cell-surface antigen profiles were assessed using the following mouse anti-human antibodies purchased from BD Biosciences: anti-CD14-FITC, anti-CD16-PE-Cy7, anti-CD80-BV421, anti-CD206-APC, and mouse anti-human CD200R-PE antibody from Invitrogen. Expression levels of M1 and M2 macrophage markers (CD80 and CD200R/CD206, respectively) were analyzed within the CD14+ population. Flow cytometry analysis was optimized by compensation, using unstained samples, isotype controls, and fluorescence minus one (FMO) controls.

### 2.7. Immunocytochemical (ICC) Analysis of CD206 Expression on Macrophage Surface

For ICC, PBMCs (3 × 10^5^) were seeded onto 96-well, µCLEAR, black plates Ref. No. 655090 (Greiner Bio-One GmbH, Kremsmünster, Austria). The process of maturation and stimulation of macrophages was conducted as described above. The average number of macrophages per well was determined during preliminary experiments. This assessment was performed by imaging of DAPI stained nuclei with the use of automated microscope Cytation™ 1 (BioTek, Agilent, Santa Clara, CA, USA) and Gen5 3.04 software. The mean number of cells per well at day 7 of culture was 3 × 10^4^. Therefore, 3 × 10^3^ MSCs per well were added to the macrophages to achieve a 1:10 ratio. Additionally, MSCs and macrophages (unstimulated, M_IFNγ_- and M2-stimulated) from each population seeded in monoculture served as controls. At the end of the experiment, cells were fixed with 4% paraformaldehyde (15 min, RT). After washing 3 times with PBS, cells were incubated with blocking buffer consisting of PBS with 2.5% donkey serum and 1% bovine serum albumin (BSA) for 30 min (RT), followed by overnight incubation (4 °C) with primary goat anti-CD206 antibody (R&D Systems, Ref. No. AF2534, 2.5 µg/mL) in blocking buffer. Next, after washing with PBS (3 times for 5 min), cells were incubated with secondary antibody (Alexa Fluor 488 donkey anti-goat IgG (H+L), Invitrogen, Waltham, MA, USA, Ref. No. A11055, dilution 1:300), Molecular Probes solution in blocking buffer for 1 h in the dark (RT). Cells were again washed 3 times for 5 min with PBS, and cell nuclei were stained with DAPI solution (20 ng/mL, for 4 min, RT). Wells were screened using a Cytation™ 1 automated fluorescent microscope (BioTek, Agilent, Santa Clara, CA, USA). Images were analyzed with Gen5 3.04 software. In the first step, MSCs nuclei were eliminated from the analysis based on data from control wells. Then, CD206 antigen expression on macrophages was assessed using the tool of the secondary mask designed around each DAPI stained nucleus. A distinction was made between total and “strong” (bright) CD206 expression using different cut-off points for mean fluorescence intensity (MFI) within the secondary mask.

### 2.8. Quantitative Assessment of the Secretory Activity of MSCs

Comparative analysis of MSCs secretory activity was performed using the Luminex method. BM-MSCs and WJ-MSCs were cultured in parallel on ø 60 mm Primaria™ cell culture dishes in standard GM (cells from 5–6 donors for each cell type were used in this assay, in some cases, cells on two subsequent passages were used, total *n* = 7 per cell type). When cells achieved approximately 90% confluence, the medium was replaced with DMEM low glucose supplemented with 4% BSA (bovine serum albumin) with or without the addition of IFNγ (25 ng/mL). After 24 h treatment, cells supernatants were collected, centrifuged at 4500 rpm for 5 min in 4 °C, transferred to new tubes, mixed, aliquoted, and frozen immediately in −80 °C. Cells were then detached from the dishes and counted to allow the amount of secreted factors to be adjusted to the number of secreting cells. All samples were analyzed at once using the custom Luminex Multiplex kit purchased in R&D Systems (Bio-Techne, Minneapolis, MN, USA), Ref. No. LXSAHM-06HGF, which contained interleukin 4 (IL-4), interleukin 6 (IL-6), interleukin 10 (IL-10), and hepatocyte growth factor (HGF) analytes. The procedure was performed according to the manufacturer’s instructions. The flow-based magnetic beads reading was performed on Luminex LX-200 Instrument. The assay was performed in duplicates.

### 2.9. The Effect of Tocilizumab on the Interaction between MSCs and Macrophages

To verify if IL-6 affects the MSC-mediated switch of macrophage phenotype to M2, the direct MSC-macrophages co-culture experiment was repeated with the addition of Tocilizumab (TCZ), a monoclonal antibody that competitively inhibits the binding of IL-6 to its receptor (IL-6R). The assay was performed on 96-well plates, and the effect was verified using immunocytochemical staining for CD206, as described above. Before co-culture, MSCs were treated for 24 h with TCZ at a concentration of 50 µg/mL (prepared from RoActemra, Roche, Basel, Switzerland, 20 mg/mL). Additionally, TCZ was added daily during 3 days of co-culture to ensure sustained blocking of IL-6R. In this part of the study, labeling of the general macrophage antigen-CD68 was additionally used in the staining procedure. For this purpose, cells were first probed with goat anti-CD206 antibody (R&D Systems Ref. No. AF2534, 2.5 µg/mL, 3 h, RT), then washed, permeabilized (blocking buffer with 0.3% (*v/v*) TritonX-100, 30 min, RT) and followed by overnight incubation (4 °C) with primary mouse anti-CD68 antibody (clone EBM11, Dako, Agilent, Glostrup, Denmark; Ref. No. M0718, dilution 1:100). After washing, a cocktail of secondary antibodies (Alexa Fluor 647 donkey anti-goat, Ref. No. A21447 and Alexa Fluor 488 donkey anti-mouse, Ref. No. A21202, both from Invitrogen, dilution 1:300) was added for 1 h, RT. Cell nuclei were stained with DAPI. In this part of the study, the expression of CD206 on macrophage surface was evaluated within cells expressing CD68 using the tool of the secondary mask designed around each DAPI stained nucleus (Gen5 3.04 software, BioTek, Agilent, Santa Clara, CA, USA). For this assay, macrophages from 3 PBMC donors and BM-MSCs from 4 donors were used (each MSC population was paired with each macrophage population). The test was performed in triplicates.

### 2.10. Trans-Well Migration Assay

The ability of the MSCs from different sources to directed, macrophage-stimulated migration was examined using trans-well inserts assay. We tested the extent to which the analyzed MSCs respond to chemotactic signals released by different subsets of macrophages. The assay was performed on 24-well Advanced TC™ plates (Greiner Bio-One GmbH, Kremsmünster, Austria, Ref. No. 662 960) using inserts with a pore size of 8 μm (Thincert™, Greiner Bio-One GmbH, Kremsmünster, Austria). Macrophages seeded onto the bottom compartment constituted the stimulating population. PBMC seeding and the process of maturation and stimulation of macrophages (into M_IFNγ_, M1, and M2) were conducted as described above. Empty wells served as unstimulated controls. MSCs were treated with IFN-γ (25 ng/mL) for 24 h before seeding on inserts. The next day, they were detached and labeled with red fluorochrome DiLC18(5)-DS (DID), Ex = 650 nm, Em = 670 nm (AAT Bioquest). The macrophages were washed, and Opti-MEM medium (Gibco, Ref. No. 11058021) supplemented with 4% FBS without the addition of cytokines was added to all wells. Did-labeled MSCs were suspended in DMEM low glucose with 4% FBS and seeded on the upper compartment (1.5 × 10^4^ cells/insert). The same number of MSCs were also seeded on the empty wells as a control to enable the estimation of the total migrating population (TMP). Each combination was seeded in duplicate. Cells were incubated at 37 °C and 5% CO_2_ for the next 48 h to allow cell migration from the inserts to the basal compartment. Afterward, inserts were removed, cells on the bottom of the wells were fixed with 4% paraformaldehyde (10 min, RT), and the cell nuclei were visualized with DAPI staining (20 ng/mL of DAPI solution for 4 min, RT). Additionally, migrating cells were trypsinized from the bottom side of inserts. They were allowed to attach for 6 h and also fixed and stained with DAPI. The cells were visualized using a Cytation™ 1 reader (BioTek, Santa Clara, CA, USA) and analyzed with Gene5 3.04 software as it was described in our previous work [20]. Briefly, the number of migrating/stimulating cells was calculated from 9 different fields of view from each well in each sample. Migrating cells were the sum of those that fell off the insert and attached to the bottom of the well during the experiment (DIDf) and those detached from the bottom of the insert (DIDins) and attached after the end of the experiment. Fields of view had the same locations in all wells and were arbitrarily selected prior to analysis. Migrating cells were identified based on the presence of DID-derived fluorescence using the tool of the secondary mask designed around each DAPI stained nucleus. Two different analyses were performed. First, the number of migrating cells for each type of stimulus was compared to the control (unstimulated migration). The sum of DIDf and DIDins was taken for this analysis and counted as the percentage of the total migrating population (to eliminate potential differences in the proliferation rate in between various MSC populations). The number of stimulating macrophages was calculated as the total number of cell nuclei (DAPI) from the analyzed area minus the number of DID+ cells. As the number of stimulating macrophages differed depending on the subpopulation and donor at the end of the experiment, for final analysis, the number of migrating MSCs (expressed as a percentage of the total number of migrating MSCs from a given population) was normalized to 1000 stimulating macrophages.

(1) $$\mathrm{migrating}\;\mathrm{potential}=\frac{\mathrm{DIDf}+\mathrm{DIDins}}{\mathrm{TMP}}\times 100\times \frac{1}{\mathrm{DAPI}-\mathrm{DIDf}}\times 1000$$

DIDf—the number of migrating cells (stained with DID) that fell off the insert.

DIDins—the number of migrating cells (stained with DID) that were detached from the underside of an insert.

DAPI—the number of total cell nuclei (stained with DAPI). Nuclei of macrophages and DIDf MSCs were counted.

TMP—total migrating population; the number of MSCs from a given population seeded at the bottom of the well in the same amount as per insert and calculated at the end of the experiment from the same arbitrary selected fields of view as in the analysis of migrating cells.

### 2.11. Cell Migration Scratch Assay—Live Cell Imaging

This assay was performed to assess how the secretome from different subsets of macrophages affects the motility of analyzed MSC types. For this assay, PBMCs were seeded on 24-well Advanced TC™ plates (Greiner Bio-One GmbH, Kremsmünster, Austria, Ref. No. 662 960). Maturation and stimulation (into M_IFNγ_, M1, and M2) of macrophages were performed as described above. After 3 days of stimulation, macrophages were washed, and Opti-MEM medium (Gibco, Ref. No. 11058021) supplemented with 4% FBS without the addition of cytokines was added to all wells. After 24 h, supernatants were collected and centrifuged to remove cells or cell fragments. MSCs were seeded onto 96-well, µCLEAR, black plates (Greiner Bio-One GmbH, Kremsmünster, Austria, Ref. No. 655090) in a concentration of 1 × 10^4^/100 µL/well. The next day, the medium with the addition of IFN-γ (25 ng/mL) was added for 24 h. Then, MSCs were then washed once, scratched in each well using a 200 µL tip and a sterile ruler to ensure a straight “wound” path. The assay was performed in triplicate. The cells were then washed once more, and the appropriate macrophages supernatants or control medium were applied to MSCs (150 µL/well). The plates were placed in a Cytation™ 1 Cell Imaging Multi-Mode Reader (BioTek, Santa Clara, CA, USA) under temperature and CO_2_ controlled conditions. The cells underwent kinetic image capturing (bright field) in a 2 h time-lapse for 24 h. Images were analyzed using Gene5 3.04 software (BioTek, Santa Clara, CA, USA). Due to the manual execution of the scratch and possible differences in the size of the wound between the wells, the degree of coverage of the scratch surface with cells was analyzed in µm^2^. For each time point, the difference between the cell-covered area at that time point and the cell-covered area at time point 0 for each well was calculated. Then, the average of 3 technical replicates was calculated. Altogether, MSCs from 4 donors (for each MSC source) and macrophages from 6 donors were used for this test. Each time, both BM-MSCs and WJ-MSCs were seeded on the same plate and exposed to supernatants of macrophages from the same donors.

### 2.12. Statistical Analysis

All the statistical analyses were performed using STATISTICA software v. 13.1 (StatSoft^®^ Polska, Krakow, Poland). First, the data distribution within groups was analyzed using the Shapiro–Wilk test. If data were compared to the control, the groups of related data with abnormal distribution were analyzed using Wilcoxon matched-pairs signed-ranks test, and groups with confirmed normal distribution were compared using Student’s T-test. Analyses of multiple groups (i.e., migration assays, differentiation of macrophages) were performed using ANOVA with Fisher’s Least Significant Difference (LSD) or Tukey post-hoc tests. Significance was set at *p* < 0.05, and graphs are presented as mean ± standard error of the mean (SEM).

## 3. Results

### 3.1. Mesenchymal Stromal Cells Identification

**Figure 1:**
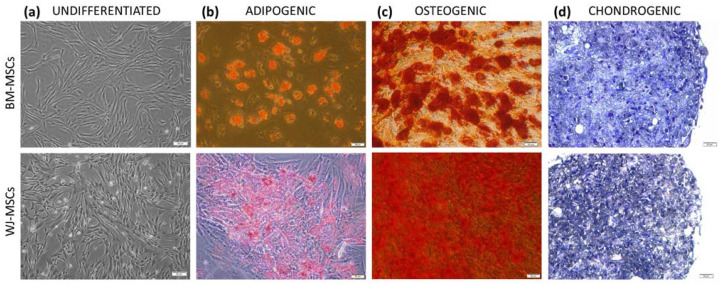
The morphology and differentiation potential of BM-MSCs (upper row) and WJ-MSCs (lower row). Light microscopy. (**a**) morphology of undifferentiated MSCs; (**b**) MSCs after adipogenic differentiation, lipid droplets stained with Oil Red O; (**c**) MSCs after osteogenic differentiation, calcium deposits stained with Alizarin Red; (**d**) MSCs after chondrogenic differentiation, proteoglycans stained with Toluidine blue; scale bars—50 µm.

Human bone marrow- and Wharton’s jelly-derived mesenchymal stromal cells were successfully isolated (each cell type from six donors). All MSC populations adhered to the surface of the plastic culture dishes forming fibroblast-like colonies. Flow cytometry analysis of cell-surface antigen profiles revealed that the mean expression of positive MSC markers amounted 98.7% for CD90, 99.4% for CD44, 99.7% for CD73 and 99.2% for CD105 in BM-MSC populations and 99.4%, 98.5%, 99.4%, and 98.8% in WJ-MSCs, respectively. The mean of 98.5% BM-MSCs and 98.9% WJ-MSCs did not express any of the negative MSC markers (CD11b, CD19, CD34, CD45, and HLA-DR). In order to confirm the ability of cells obtained from both sources to differentiate into osteocytes, chondrocytes, and adipocytes, specific staining was successfully performed (Figure 1).

### 3.2. BM- and WJ-MSCs Inhibit Proliferation of Allo-Stimulated Lymphocytes in a Similar Extent

**Figure 2:**
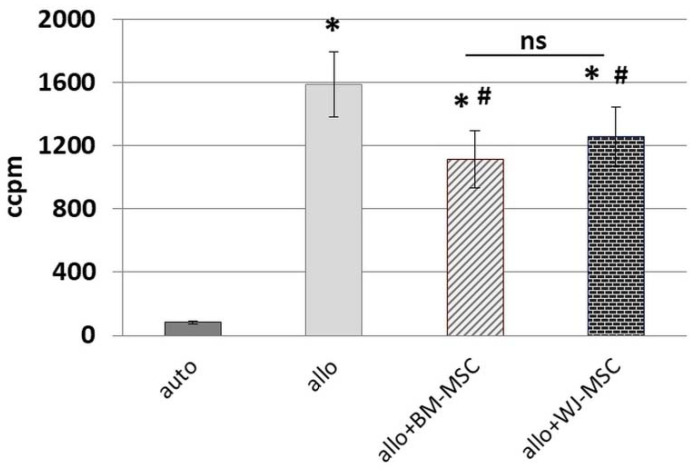
The effect of BM-MSCs and WJ-MSCs on the allo-stimulated lymphocytes proliferation. The graph presents the results of the Mixed lymphocytes reaction (MLR) assay. Auto—the proliferation of lymphocytes stimulated with irradiated lymphocytes from the same donor. Allo—proliferation of lymphocytes stimulated with irradiated lymphocytes from the unrelated donor. Data presented as means (± SEM). * *p* < 0.001, in comparison to the “auto” group (T-test for related data); # *p* < 0.01, comparison to the “allo” group (T-test for related data). The effect of different MSC types on lymphocytes was compared using the U Mann–Whitney test. ns—statistically not significant. PBMCs from 10 donors were used (5 pairs), each combined with one BM-MSCs (*n* = 5) and one WJ-MSCs (*n* = 5) population. Total *n* = 10 for each MSC type.

In the first step, we aimed to confirm the inhibitory effect of MSCs on activated lymphocytes and to compare the effect of analyzed MSC types. The addition of irradiated PBMCs from donor A to PBMCs from unrelated donor B resulted in expected intensive stimulation and proliferation of lymphocytes (*p* < 0.001 in comparison to auto-stimulated cells) (Figure 2).

The addition of MSCs to such a mixture (at an MSCs:PBMCs ratio of 1:10) significantly inhibited the proliferation of stimulated lymphocytes. This effect was observed regardless of the MSC source (*p* < 0.001 for both groups). To compare the magnitude of the effect of MSCs on lymphocytes, the difference (“Δ”) between proliferation after allo-stimulation with and without the addition of MSCs was determined, and then the “Δ” values for both types of MSC were compared as independent data (U Mann–Whitney test). The difference between the effects of analyzed MSC types on lymphocytes was not statistically significant (*p* > 0.05).

### 3.3. The Effect of IFN-γ Stimulation on the Secretion of Selected Factors by Human BM- and WJ-MSCs

**Figure 3:**
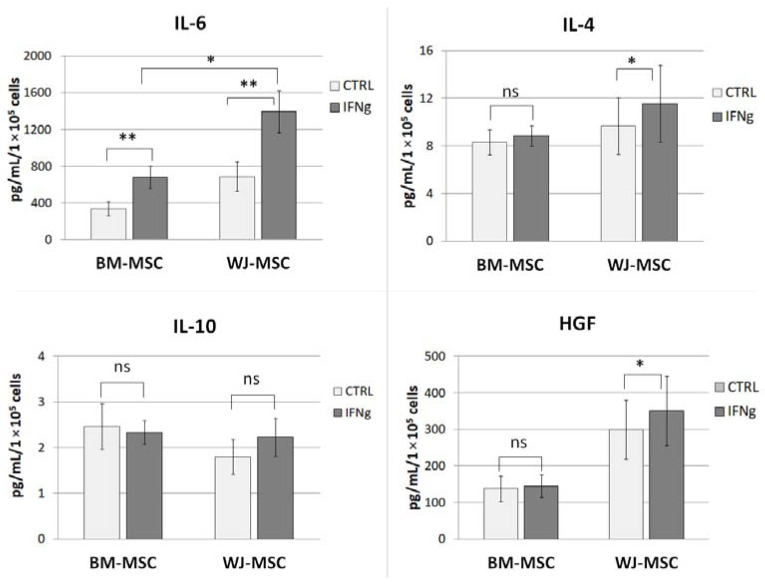
The effect of IFN-γ stimulation on the secretion of selected factors by human BM- and WJ-MSCs by Luminex assay. Bar graphs represent the mean ± SEM concentration of factors in the supernatant adjusted to 1 × 10^5^ secreting cells. The comparisons of control (CTRL) vs. IFNγ-stimulated MSCs (IFNγ) were conducted using Wilcoxon test or T-test for related data (depending on the data distribution, which was determined using Shapiro–Wilk test); the differences between BM-MSCs and WJ-MSCs were analyzed using U Mann–Whitney test or T-test for unrelated data (depending on the data distribution). The normality was checked using the Shapiro–Wilk test. * *p* < 0.05; ** *p* < 0.01; ns—statistically not significant; *n* = 7 in each group, BM-MSCs from 6 donors and WJ-MSCs from 5 donors were used for this assay.

To confirm the secretory activity of isolated MSCs, their response to pro-inflammatory stimulation and to conduct a comparative analysis of the paracrine potential of BM- and WJ-MSCs, we have analyzed the level of selected cytokines/growth factors in the supernatants using the Luminex method. For this assay, we selected secretory factors that were previously shown to be important in the interaction between MSCs and macrophages: IL-4, IL-6, IL-10, and HGF. As WJ-MSCs are known to display a higher proliferation rate than BM-MSCs, cells were detached and counted after supernatant collection. The mean number of WJ-MSC_CTRL_ was 30% higher than the number of BM-MSC_CTRL,_ and the mean number of WJ-MSC_IFNγ_ was 48% higher than BM-MSC_IFNγ_. Although these differences were not statistically significant (*p* = 0.14 and *p* = 0.10, respectively), we decided to take into account the number of secreting cells before further statistical analysis, so the concentration of the analyzed factors was calculated per 10 × 10^5^ cells. Stimulation with IFNγ significantly increased the level of IL-6 secreted by both types of MSCs (fold change = 2.01 for BM-MSCs and 2.02 for WJ-MSCs, *p* < 0.01 for both types of MSCs) (Figure 3).

Moreover, after IFNγ stimulation, the secretion of this cytokine by WJ- MSCs was significantly higher compared to that of BM-MSCs (*p* = 0.02, Figure 3). The addition of IFNγ also altered the secretion of IL-4 by BM-MSCs (by 19% compared to control) and HGF by WJ-MSCs (by 17% compared to WJ-MSC_CTRL_), both *p* < 0.05. There were no significant differences in the secretion of IL-10 by MSCs in response to IFNγ stimulation.

### 3.4. BM- and WJ-MSCs Drive Macrophages into M2 Phenotype in a Similar Extent

**Figure 4:**
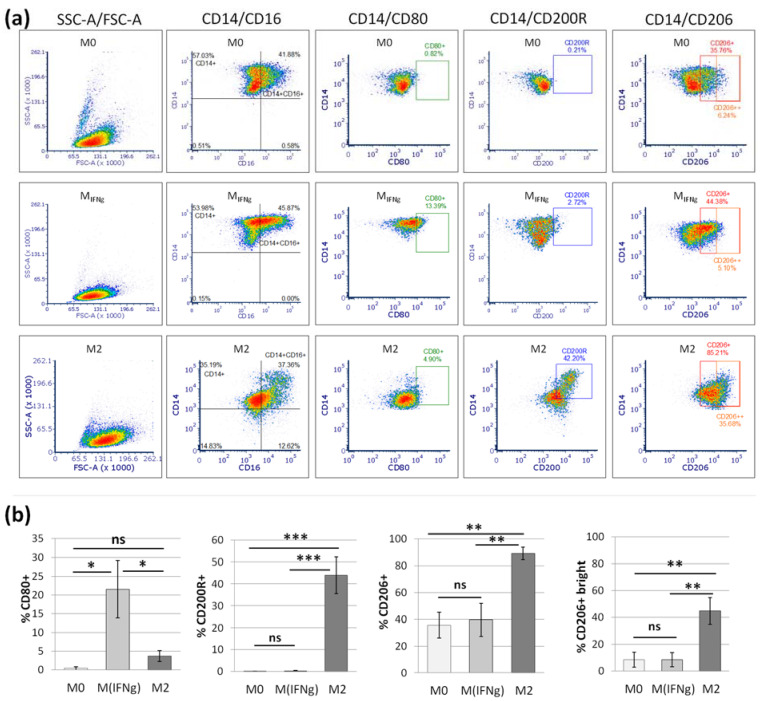
The phenotype of differentially stimulated macrophages. The graph shows the results of the flow cytometry analysis. (**a**) Representative density plots illustrating the expression of different markers on unstimulated (M0), IFNγ stimulated (M_IFNγ_) and IL-4/IL-10 stimulated (M2) macrophages. Numbers on the density plots indicate percentage (%) of cells positive for certain markers. (**b**) Bars represent the mean (± SEM) expression of markers for different subsets of macrophages. Data analyzed using one-way ANOVA with post-hoc Tukey test. * *p* < 0.05, ** *p* < 0.01, *** *p* < 0.001; ns—statistically not significant; *n* = 7.

To perform a comparative analysis of the effect of MSCs on macrophages, the phenotype of macrophages treated with different cytokines was first assessed (Figure 4a). Cytometric analysis showed that the IFNγ-treated macrophages (M_IFNγ_) displayed significantly higher CD80 expression than unstimulated (M0) or IL-4/IL-10 stimulated (M2) macrophages (*p* < 0.05) (Figure 4b).

Among macrophages stimulated to the M2 phenotype, an average of 43.9% acquired CD200 receptor expression, while M0 and M_IFNγ_ macrophages did not have this marker on the surface at all (*p* < 0.001 between M2 and other subtypes of macrophages). On average, 89% of M2 macrophages expressed the CD206 marker, but this molecule was also present on a substantial number of M0 and M_IFNγ_ cells, averaging 35.5% and 39.4%, respectively. To better exploit the differentiating nature of the CD206 antigen, we further assessed the percentage of cells with strong CD206 expression, CD206++. On average, 44.8% of M2 macrophages displayed strong CD206 expression, while for M0 and M_INFγ,_ it was 8.6% and 8.5%, respectively (*p* < 0.01 between M2 and other subtypes of macrophages) (Figure 4b).

**Figure 5:**
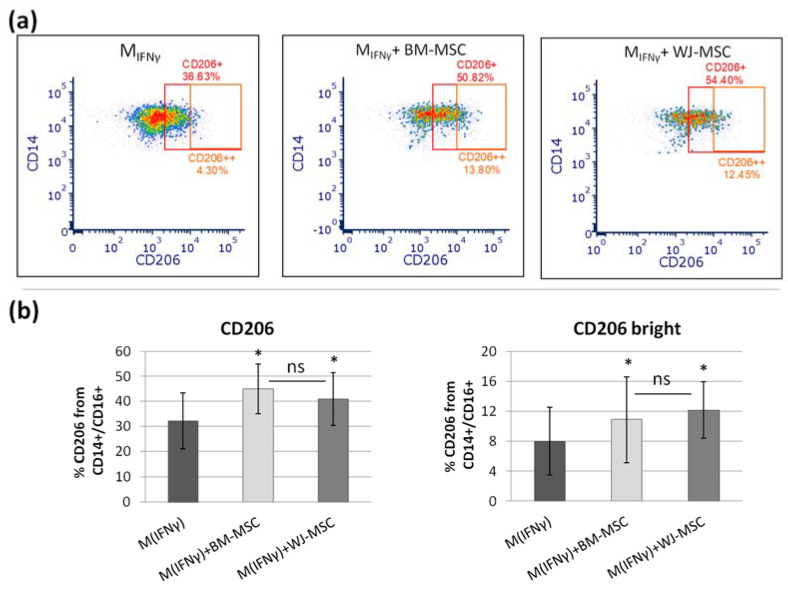
The effect of BM-MSCs and WJ-MSCs on the differentiation of macrophages. The graph shows the results of the flow cytometry analysis. (**a**) Representative density plots illustrating the expression of CD206 antigen on IFNγ-stimulated macrophages (M_IFNγ_) and the same macrophages after addition of bone marrow (BM) mesenchymal stromal cells (MSCs) and Wharton jelly (WJ)-MSCs. Numbers above gates indicate percentage (%) of cells positive for CD206, and numbers below gates indicate percentage of cells with strong expression of CD206 (CD206++). (**b**) The mean (± SEM) expression of CD206 (and CD206++) of M_IFNγ_ cultured in monoculture and in co-culture with BM-MSCs and WJ-MSCs. The effect of different types of MSCs on M_IFNγ_ analyzed using Wilcoxon test in comparison to the same macrophages cultured in monoculture, * *p* < 0.05, *n* = 8. The normality was checked using the Shapiro–Wilk test. The comparison of effect of the different types of MSCs was determined as the difference (“Δ”) between CD206 expression after co-culture with MSCs with and the expression without the addition of MSCs, and then the “Δ” values for both types of MSCs were compared as independent data (U Mann–Whitney test). ns—statistically not significant. For this assay, macrophages from 6 PBMC donors were used. Every population was paired in parallel with BM-MSCs and WJ-MSCs populations; WJ-MSCs from 4 donors and BM-MSCs from 5 donors were used.

After confirming the ability to distinguish between different macrophage subpopulations (Figure 4), the effect of the analyzed MSCs types on macrophage differentiation was investigated, and a comparative analysis was performed. The addition of MSCs to macrophages (3 days of direct co-culture in an approximate ratio of 1:10) resulted in a significant increase in the expression of the M2 phenotype marker, CD206. After addition of BM-MSCs, the percentage of CD206+ macrophages increased from a mean of 32.2% to 45% (*p* = 0.003, Wilcoxon test) and after addition of WJ-MSCs the percentage of CD206+ macrophages amounted 40.1% (*p* = 0.016 in comparison to control—M_IFNγ_) (Figure 5).

The percentage of macrophages with strong CD206 expression after the addition of BM-MSCs and WJ-MSCs increased from 8% to 12.1% and 10.9%, respectively (both *p* < 0.05). The difference between the effects of adding different types of MSCs was not statistically significant. The expression of CD200R on macrophages was not significantly affected by the co-culture with MSCs, regardless of MSC origin.

**Figure 6:**
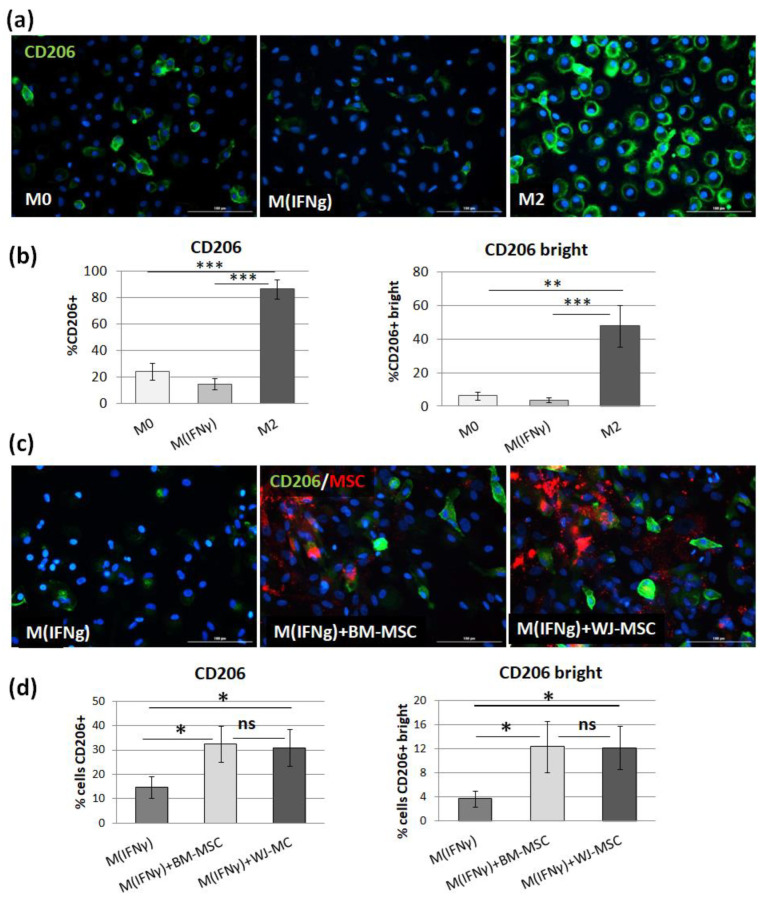
The effect of BM-MSCs and WJ-MSCs on the differentiation of macrophages into M2 phenotype. (**a**) Representative images illustrating the CD206 expression in unstimulated (M0), IFNγ-stimulated (M_IFNγ_), and IL-4/IL-10 stimulated (M2) macrophages detected by immunocytochemistry assay using an anti-CD206 antibody. The signal was developed using green AF488-conjugated secondary antibody, and nuclei were counterstained with DAPI (blue). The scale bars indicate 100 μm. (**b**) Bar graphs (mean ± SEM) represent the quantitative analysis of CD206 expression in different macrophage subpopulations as a percentage of all cells. Data collected from four experiments performed in duplicates with two PBMCs donors in each experiment (*n* = 8). Data analyzed using one-way ANOVA with post-hoc Tukey test. ** *p* < 0.01, *** *p* < 0.001; (**c**) Representative images illustrating the CD206 expression in M_IFNγ_ macrophages without or with the addition of MSCs of different origin (BM—bone marrow and WJ—Wharton’s jelly). The CD206 molecule stained in green, MSCs stained in red (DID, 1,1′-Dioctadecyl-3,3,3′,3′-tetramethylindodicarbocyanine-5,5′-disulfonic acid), nuclei are blue. The scale bars indicate 100 μm. (**d**) The mean (± SEM) expression of CD206 (and CD206 bright) of M_IFNγ_ cultured in monoculture and in co-culture with BM-MSCs and WJ-MSCs. The effect of different types of MSCs on M_IFNγ_ was analyzed using Wilcoxon test or T-test for related data (depending on the data distribution, which was determined using Shapiro–Wilk test) in comparison to the same macrophages cultured in monoculture, * *p* < 0.05, *n* = 8 (for both BM-MSCs and WJ-MSCs, cells from four donors, and macrophages from eight PBMCs donors were used). The comparison of effect of the different types of MSCs was determined as the difference (“Δ”) between CD206 expression after co-culture with MSCs and the expression without the addition of MSCs, and then the “Δ” values for both types of MSCs were compared as independent data (U Mann–Whitney test). ns—statistically not significant.

Considering the technical difficulties related to the detachment of macrophages from the culture surface for cytometric analysis, we implemented another method to verify these results. We performed analogous experiments (the direct co-culture of macrophages with MSCs), and we determined the effect of MSCs on the differentiation of macrophages using the immunocytochemistry technique, which does not require the detachment of macrophages for final evaluation. First, the reliability of the ICC method for this kind of assessment was checked by analyzing the expression of the CD206 marker on unstimulated, IFNγ-stimulated, and IL-4/IL-10-stimulated macrophages. The expression of CD206 antigen was present on 24.4%, 14.7%, and 86.5% of M0, M_IFNγ,_ and M2 macrophages, respectively (*p* < 0.001 between M2 and other subtypes of macrophages, one-way ANOVA with post-hoc Tukey test) (Figure 6).

Additionally, the percentage of macrophages with strong expression of CD206 was assessed, and it amounted 2.46%, 1.34%, and 12.51% for M0, M_IFNγ,_ and M2 macrophages, respectively (*p* < 0.01 between M2 and other subtypes of macrophages). Addition of MSCs to direct co-cultures for 3 days resulted in a significant increase in CD206 expression on IFNγ-stimulated macrophages from a mean of 14.7% CD206+ cells to 32.5% for BM-MSCs and 30.9% for WJ-MSCs (both *p* = 0.01 in comparison to the control M_IFNγ_). The percentage of cells with strong CD206 expression also significantly increased from 3.75% to 12.4% (*p* = 0.03) and to 12.2% (*p* = 0.01) after addition of BM-MSCs and WJ-MSCs, respectively. The difference between the effects of adding different types of MSCs was not statistically significant.

### 3.5. Tocilizumab Does Not Alter the Effect of MSCs on the Differentiation of Macrophages

**Figure 7:**
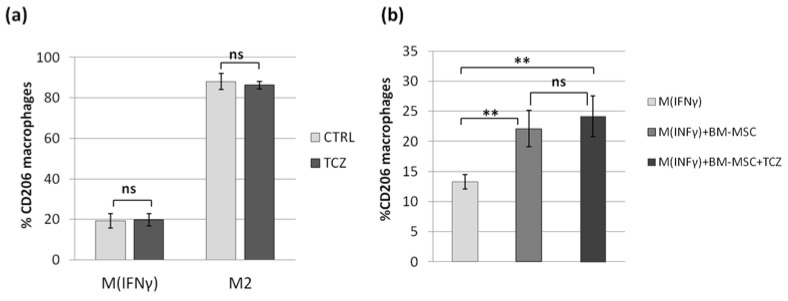
The effect of IL-6 receptor blockade with Tocilizumab on MSCs-related macrophage differentiation into the M2 phenotype. Immunocytochemical analysis. Bars represent the mean percentage of CD206 expressing cells within CD68+ cells (macrophages). (**a**) The effect of TCZ on CD206 expression in M_IFNγ_ and M2 macrophages in monoculture (*n* = 6); (**b**) The effect of TCZ on CD206 expression in M_IFNγ_ cultured in co-culture with BM-MSCs for 3 days (*n* = 12, macrophages from 3 donors and MSC from 4 donors were used in this experiment). Data analyzed using the Wilcoxon test or T-test for related data (depending on the data distribution, which was determined using the Shapiro–Wilk test) ** *p* < 0.01 For the comparison of the effect of MSCs with and without TCZ on CD206 expression, the difference (“Δ”) between CD206 expression after co-culture with MSCs (with or without TCZ) and the expression without the addition of MSCs, and then the “Δ” values for treated and untreated MSCs were compared as independent data (U Mann–Whitney test). ns—statistically not significant.

The Luminex analysis revealed that BM-MSC_IFNγ_ secrete significantly less IL-6 than WJ-MSC_IFNγ_, and on the other hand, the effect of both MSCs types on the differentiation of macrophages did not differ significantly. These results suggested that IL-6 is not a key molecule that drives the MSC-mediated switch of macrophages into M2 phenotype in the direct co-culture system used in this study. To verify this assumption, we tested the effect of an anti-IL-6R antibody (Tocilizumab, TCZ) on MSCs-related macrophage differentiation. The strong inhibitory effect of TCZ (50 µg/mL) on the activation of IL-6 signaling pathways in human MSCs was confirmed in a preliminary experiment by analyzing the level of phosphorylated STAT3 protein by Western blot (data not shown). Then, the effect of TCZ on different subsets of macrophages was evaluated in monoculture. The three-day incubation with TCZ had no effect on the CD206 marker expression (Figure 7a).

Next, the MSC-M_INFγ_ co-culture with or without the addition of TCZ was carried out for 3 days. The addition of TCZ to the MSC-macrophages co-culture did not significantly affect the impact of MSCs on the switch of macrophages into the M2 phenotype measured on the basis of CD206 expression (Figure 7).

### 3.6. Effect of Macrophages on the Migratory Ability of BM-MSCs and WJ-MSCs—Chemoattraction and Mobility

**Figure 8:**
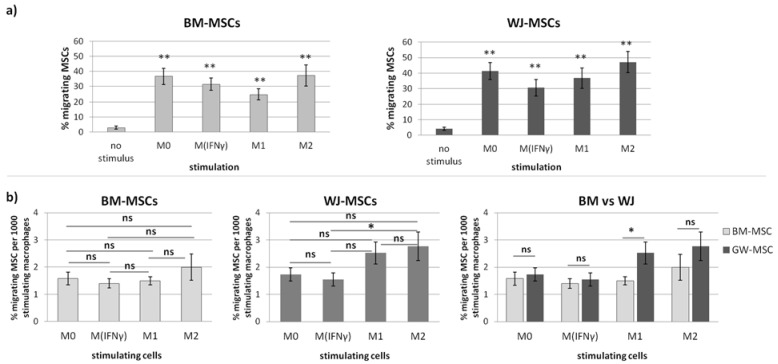
The effect of different subsets of macrophages on the chemotactic activity of human BM-MSCs and WJ-MSCs assessed using the trans-well migration assay (8 µm). (**a**) The migrating BM-MSCs and WJ-MSCs stimulated by different subsets of macrophages (M0—unstimulated macrophages, M_IFNγ_—IFNγ stimulated, M1—IFNγ/LPS-stimulated, and M2- IL-4/IL-10 stimulated) macrophages in comparison to the unstimulated control (no stimulus—migration to the empty well). Bars represent the mean percentage (± SEM) of MSCs found in the lower compartment after 48 h (with respect to the number of cells seeded in the upper compartment at the start of the experiment); Data analyzed using T-test for related data in comparison to unstimulated control; ** *p* < 0.01. (**b**) The comparison of different subsets of macrophages as stimulants of MSCs chemotaxis. The data presented as the mean percentage (± SEM) of MSCs found in the lower compartment after 48 h (with respect to the number of cells seeded in the upper compartment at the start of the experiment) per 1000 stimulating cells. Within one MSC type, data were analyzed using one-way ANOVA with Tukey post-hoc correction. The comparison of BM-MSCs and WJ-MSCs within one stimulus was performed using a T-test for independent data (normal data distribution in all groups confirmed with Shapiro–Wilk test). * *p* < 0.05; ns—statistically not significant. Data collected from 4 independent experiments, total *n* = 12.

To evaluate the extent to which different subsets of macrophages attract MSCs from various sources, migration tests were employed. First, a trans-well assay (8 µm pore size) was performed where different subsets of macrophages constituted the source of stimuli for chemotaxis of BM- and WJ-MSCs. The results indicate that macrophages, regardless of their phenotype, are very potent chemotactic stimulators for MSCs. On average, only 2.8% BM-MSCs and 4.2% WJ-MSCs of the total number of cells initially seeded to the upper compartment were found in the lower compartment of the well after 48 h if no stimulation was applied (only medium containing 4% FBS) (Figure 8a).

This basal rate of migration did not differ significantly between BM-MSCs and WJ-MSCs (*p* > 0.05) (Figure 8a). If macrophages (M0, M_IFNγ_, M1, or M2) were used as stimulants, the percentage of migrating BM-MSCs increased to 36.6%, 31.4%, 24.8%, and 37%, respectively (*p* < 0.01 for all groups in comparison to the unstimulated control). The percentage of WJ-MSCs in parallel experiments increased to 41.3%, 30.5%, 36.7%, and 47.1%, respectively (*p* < 0.01 for all groups in comparison to the unstimulated control) (Figure 8a). In the second step, different subsets of macrophages were compared among each other as stimulants of MSCs. In this analysis, the number of stimulating macrophages was additionally taken into account. For both BM-MSCs and WJ-MSCs, M2 appeared to be the strongest chemoattractant among analyzed subsets of macrophages (Figure 8b). However, in the case of BM-MSCs, no statistically significant differences were observed between groups (Figure 8b). In the case of WJ-MSCs, M2 was found to be a significantly stronger chemoattractant than M_IFNγ_ macrophages (*p* = 0.02). No other significant differences between groups were noted in regards to WJ-MSCs (Figure 8b). Finally, the relative migration of BM-MSCs and WJ-MSCs under stimulation with different subsets of macrophages was compared. This analysis showed that WJ-MSCs were significantly more prone to respond to chemotactic signals released by M1 macrophages than BM-MSCs (1.7-fold difference, *p* < 0.04, Figure 8b). The other subsets of macrophages attracted BM-MSCs and WJ-MSCs to a similar extent.

**Figure 9:**
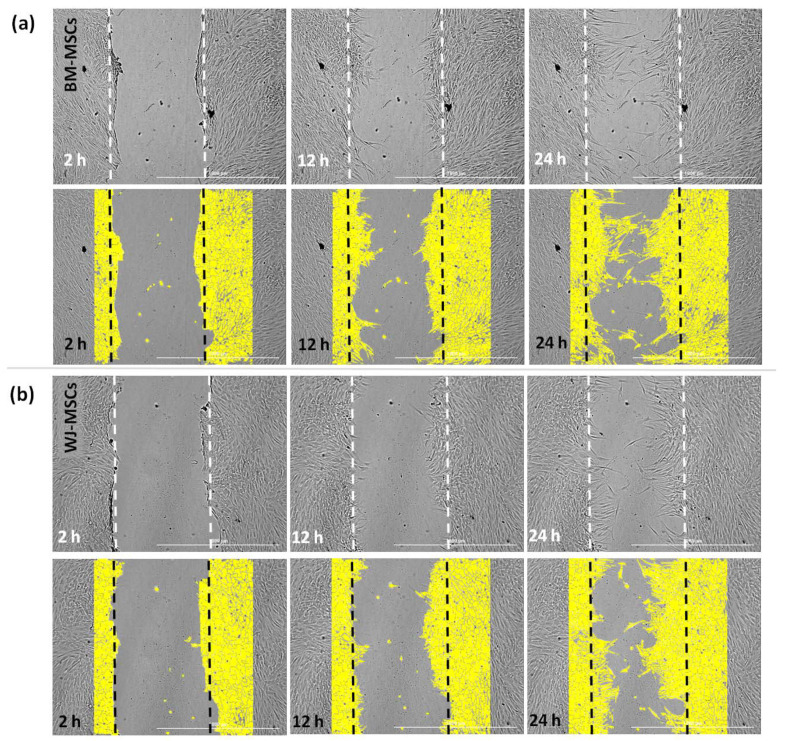

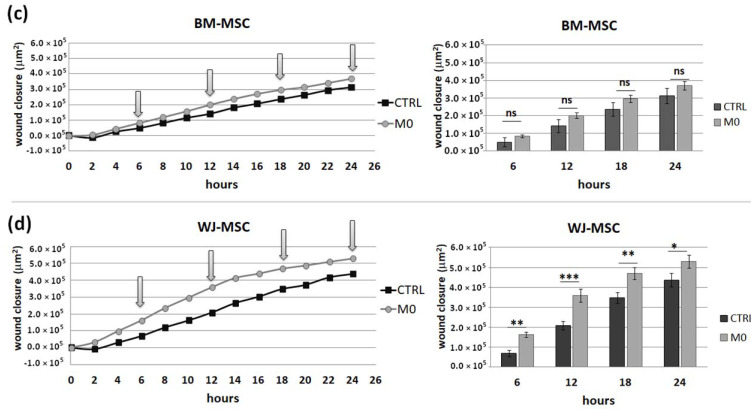
The effect of secretome from M0 macrophages on the mobility of human BM-MSCs and WJ-MSCs. The scratch assay. Images representing the behavior of mesenchymal stromal cells from (**a**) bone marrow and (**b**) Wharton’s jelly during a scratch test and the way of analysis. Upper row—pictures of the same field at different time points; lower row—pictures of the same fields of view with the area occupied by the migrating cells marked by the software (yellow color). The “wound” closure was documented every 2 h for 24 h, and statistical analysis was performed at 6, 12, 18, and 24 h time points (arrows online charts). Scratch covering presented in µm^2^ in time (h) in relation to the time point 0. The scale bar indicates 1000 μm. Migration of BM-MSCs (**c**) and WJ-MSCs (**d**) analyzed in medium supplemented with 4% of FBS (CTRL) or in the presence of conditioned medium from M0 macrophages (M0). Bars represent mean values (± SEM). Data analyzed using Wilcoxon test or T-test for related data (depending on the data distribution, which was determined using Shapiro–Wilk test) * *p* < 0.05; ** *p* < 0.01; *** *p* < 0.001; ns—statistically not significant. Data collected during 3 independent experiments, MSCs from 3 different donors used for each MSC type, macrophages from 6 different donors used, *n* = 6.

Additionally, we examined how the macrophage-derived soluble factors affect the general mobility of MSCs. For this purpose, a scratch assay was performed in which the “wound” closure was documented every 2 h for 24 h, and statistical analysis was performed at 6, 12, 18, and 24 h time points. The automated microscope and software used allowed for successful analysis of the process of migration (Figure 9a).

First, the migration of MSCs was assessed under baseline conditions and in the presence of a conditioned medium from M0 macrophages (CM-M0). The results indicate that CM-M0 did not affect the mobility of BM-MSCs (*p* > 0.05 at all analyzed time points, Figure 9b), while WJ-MSCs increased their mobility under the influence of factors secreted by macrophages. At all time points, wound closure was significantly higher under CM-M0 than under baseline conditions (Figure 9c).

**Figure 10:**
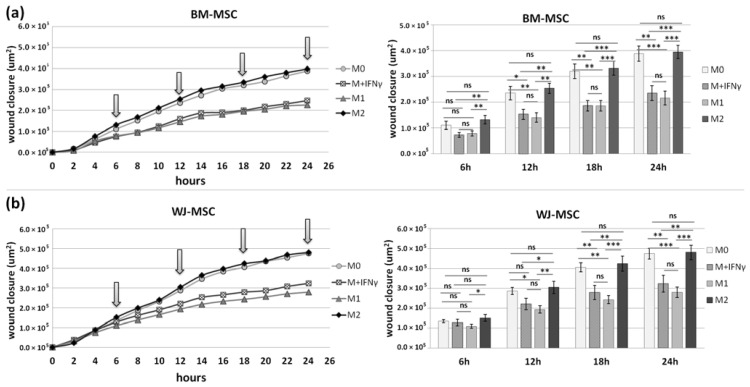
The effect of different subsets of macrophages on the mobility of human BM-MSCs (**a**) and WJ-MSCs (**b**). The scratch assay. The “wound” closure was documented every 2 h for 24 h, and statistical analysis was performed at 6, 12, 18, and 24 h time points. Scratch covering presented in µm^2^ in time (h) in relation to the time point 0. MSCs migrated in the presence of conditioned medium from M0 macrophages (M0), IFNγ-stimulated macrophages (M_IFNγ_), IFNγ/LPS-stimulated macrophages (M1), and IL-4/IL-10-stimulated macrophages (M2). Bars represent mean values (± SEM). Data were analyzed using one-way ANOVA with Tukey post-hoc correction. * *p* < 0.05; ** *p* < 0.01; *** *p* < 0.001; ns—statistically not significant. Data collected during 3 independent experiments, MSCs from 4 different donors used for each MSC type, macrophages from 5 different donors used, *n* = 10.

Next, we investigated how different subsets of macrophages affect the mobility of MSCs. The results showed a very similar response pattern in both BM-MSCs and WJ-MSCs mobility. Wound closure was significantly slower under the influence of M_IFNγ_ and M1 macrophages than under the influence of M0 and M2 macrophages regardless of MSC source, which was particularly pronounced at the 18 and 24 h time points (Figure 10).

## 4. Discussion

The ability of MSCs to modify immune processes is of particular interest to researchers and clinicians [21]. Currently, there are over 1000 MSC-related clinical trials registered on the NIH Clinical Trial Database (https://clinicaltrials.gov/, access on 25 August 2021), and a substantial number of these registered studies are or were being conducted for immune- or inflammation-mediated diseases such as autoimmune diseases, graft versus host disease (GVHD), or, more recently, for COVID-19. However, it is clear that many aspects of MSCs transplantation require clarification and further development. Among them are issues such as a better understanding of the immunomodulatory activity of MSCs and identifying the differences (or similarities) between MSCs from different tissues, which may allow for the best match of MSC type to specific disease states. Interestingly, among the currently registered clinical trials—there are a similar number of studies using mesenchymal stromal (stem) cells from bone marrow and from the umbilical cord (Wharton’s jelly). This indicates the need for studies that directly compare the properties of cells from these two different sources.

The best recognized and probably the most important from a clinical point of view is the influence of MSCs on lymphocytes. In regards to this point, several studies that directly compared the potential of MSCs from different sources have already been published (reviewed in the work of [7]). Of the four studies directly comparing the effects of BM-MSCs and WJ-MSCs on lymphocytes, two of them indicated greater suppressive activity of WJ-MSCs [5,22], while the remaining two indicated no significant differences between the effects of the two types of MSCs on lymphocytes [23,24]. In our study, we also performed such a comparative experiment. In view of inconsistency in previously published data, we aimed to maximally strengthen the reliability of our results. As reported in the literature, the immunomodulatory properties of MSCs may vary between donors [25]. To minimalize the effect of this factor, we used cells from five different donors for each type of MSC, and each population was tested with PBMCs from two different donors. Each time, the same PBMCs were cultured in parallel with the BM-MSC and WJ-MSC populations. We have confirmed the inhibitory effect of both BM-MSCs and WJ-MSCs on the proliferation of allo-activated lymphocytes. The magnitude of this influence did not differ significantly between analyzed MSC types, which indicates that both BM-MSCs and WJ-MSCs can suppress lymphocyte proliferation to a similar extent.

It is clear that the inhibitory effect of MSCs on lymphocytes seems to be crucial in the immunomodulatory activity of this population. Nevertheless, recently, increasing attention has been paid to interactions between MSCs and cells of the innate immune system, especially macrophages. It has been suggested that macrophage-mediated activity may be one of the important mechanisms of the therapeutic effect of MSCs in autoimmune diseases [26]. Previously, it was shown that macrophages are driven into an M2-like phenotype by human MSCs isolated from different sources: bone marrow [10,25,27], umbilical cord (Wharton’s jelly) [28,29], placenta [30], or adipose tissue [31]. However, according to our knowledge, none of the previously published reports directly compared MSCs from two different sources regarding their ability to affect macrophages. In our study, we aimed to perform such a direct comparison. First, we evaluated the secretory activity of isolated BM-MSCs and WJ-MSCs in terms of factors that are known to be involved in the alternative macrophage activation (IL-4 and IL-10) [17] and that were postulated to be involved in the MSC-macrophages interaction (IL-6 and HGF) [29,32,33]. Our results are consistent with other reports on the overall secretory activity of MSCs in terms of selected factors, secreting IL-6 most abundantly, followed by HGF, moderate amounts of IL-4, and very low amounts of IL-10 [19,29]. In unstimulated MSCs, none of the analyzed factors differed significantly between BM-MSCs and WJ-MSCs (concentration of factors adjusted to the number of secreting cells). Stimulation of MSCs with a pro-inflammatory factor (IFNγ) resulted in a slightly different response of BM-MSCs and WJ-MSCs. Previously, Prasanna et al. [22] reported that human WJ-MSCs and BM-MSCs respond differently to pro-inflammatory stimulation. Our results indicate that WJ-MSCs are more prone to respond to IFNγ stimulation than BM-MSCs because they significantly increased IL-4 and HGF secretion, while BM-MSCs did not. Moreover, after stimulation, the concentration of secreted IL-6 was significantly higher in supernatants from WJ-MSCs than in those produced by BM-MSCs (2.04-fold, *p* < 0.05). Since IL-6 was postulated to play an important role in the effects of MSCs on macrophages [29], this difference seemed worthy of further investigation.

In the next step, we tested to what extent BM-MSCs and WJ-MSCs affect the phenotype of macrophages. For this experiment, macrophages were stimulated into M1-like phenotype, as it was previously demonstrated that the effect of MSCs on M1 macrophages is more prominent than on unstimulated or M2 macrophages [27]. Moreover, in the case of using MSCs as a therapeutic agent, the aim will be to limit the existing inflammation, thus affecting mainly the pro-inflammatory M1 macrophages. In the majority of the experiments in this study, we induced macrophages into an M1-like phenotype by stimulation with IFNγ in a concentration of 10 ng/mL. Although the classical in vitro activation into M1 phenotype include using both IFNγ and LPS, Abumaree et al. [34] demonstrated that the stimulation with IFNγ only also induced the expression of markers strictly associated with M1 macrophages, such as CD80, which was also confirmed in the present study. Next, using the flow cytometry (FC) method, we have confirmed previous reports that MSCs stimulate macrophage differentiation toward the M2-like phenotype, which was proved by a statistically significant increase in CD206 protein expression within the CD14+ population after 3 days of direct co-culture with MSCs compared to the same macrophages cultured without addition of MSCs. The CD206 is a well-recognized marker of the M2 population [35,36], and based on previous reports. This factor most consistently indicated the effect of MSC on the macrophage population [10,25,27,28,30,32]. The shift toward the M2 phenotype was observed regardless of MSC type, and the magnitude of the effect driven by BM-MSCs and WJ-MSCs did not differ significantly between each other. To increase the reliability of our data, we used another technique to confirm obtained results. It is known that primary human macrophages during culture become firmly adhered to the culture surface and their detachment for the analysis in an unaffected form is a challenge [37]. Therefore, we introduced an immunocytochemical (ICC) method to assess the status of macrophages. First, we demonstrated that the applied ICC method can effectively distinguish unstimulated (M0), IFNγ-stimulated (M_IFNγ_), and IL-4/IL-10-stimulated (M2) macrophages. An automated microscope (Cytation™ 1; BioTek, Agilent, Santa Clara, CA, USA) allowed for the documentation of images from previously determined fields of view, the same for all wells, which ensured the objectification of the obtained data. The evaluation of CD206 expression on macrophages using the ICC method after co-culture with MSCs confirmed the results obtained with the FC method—both BM-MSCs and WJ-MSCs significantly induced the differentiation of macrophages toward M2 phenotype (*p* < 0.05), and there was no significant difference in this effect between two analyzed MSC types (*p* > 0.05). The main advantage of the proposed analysis of macrophages using ICC is that it does not require the detachment of cells from the culture dish prior to analysis, and therefore their phenotype can be evaluated in their real actual status. The disadvantage, however, is that it is very difficult to reliably assess multiple antigens simultaneously using ICC, which is a standard in the flow cytometry method. Nevertheless, in one experimental set (the part using TCZ), we additionally successfully stained CD68 antigen for ICC to ensure that only macrophages were considered. Therefore, we claim that in some cases, the ICC method can be used alternatively to FC in order to assess the changes in the phenotype of macrophages. Altogether, our results from this part of the study indicate that both hBM-MSCs and hWJ-MSCs shift macrophages toward M2-like phenotype to a similar extent.

In view of the results described above, we decided to test the role of IL-6 in the MSCs-macrophages interaction. Previously, Pilny et al. demonstrated the importance of IL-6 in the influence of hAT-MSCs on the polarization of macrophages in a murine model of limb ischemia [32]. In our study, stimulated WJ-MSCs secreted significantly more IL-6 than BM-MSC, but in functional analysis, WJ-MSCs did not show a greater effect on macrophages than BM-MSCs. This inconsistency led us to perform an additional experiment in which the effects of MSCs on macrophages were tested in the presence of an antibody against the IL-6 receptor (TCZ), thereby preventing signal transduction from this cytokine. In this experiment, we again confirmed the effect of MSCs on the macrophage phenotype; however, we also showed that blocking IL-6 signaling did not attenuate the observed interaction. These results were not consistent with the conclusions presented by Pilny et al. [29]. However, this group used MSCs from adipose tissue, while another study [33] indicated that IL-6 may be a more important mediator in MSC-macrophage interactions when using AT-MSCs than BM-MSCs (WJ-MSCs were not analyzed in that study). It is additionally known that more than one factor is involved in the effects of MSCs on monocytes/macrophages [38]. It is not fully understood how different mediators interact with each other—they may have a complementary effect, but some data indicate that there is also an overlapping effect [29], so excluding one factor does not necessarily have a significant effect on the functional result, even if that factor does play a significant role.

In the second part of this study, we aimed to determine the effect of macrophages on the migratory activity of MSCs with a direct comparison of BM-MSCs and WJ-MSCs. It is important to remember that both MSCs and macrophages show high plasticity and, therefore, when combined together, form a very dynamic system that is difficult to analyze. Previously, it was already pointed out in the literature that MSC-macrophage interaction has a reciprocal nature [14,39,40,41]. For example, it was demonstrated that direct cell-to-cell interaction (via the CD54 molecule) between macrophages and MSCs not only affects the phenotype of macrophages but also increases the immunosuppressive properties of MSCs [40]. This finding may have important implications for the therapeutic effect of MSCs in patients with immune-mediated or inflammatory diseases. However, to enable this potentially beneficial interaction, first of all, transplanted MSCs and tissue-resident macrophages have to meet each other. It is well known that bringing MSCs into their target site (both after systemic and local administration) is one of the challenges of MSC-based therapies [15,16]. It is clear that getting MSCs to their destination is dependent on chemotactic signals, the ability to respond to them, and the overall mobility of the migrating population. Macrophages, as tissue-resident cells concentrated in inflamed sites, certainly play a role in the migration of MSCs to their target sites. However, studies evaluating this type of interaction are lacking in the literature. Therefore, we were particularly interested in how macrophages affect both the chemotaxis and overall mobility of MSCs, whether this effect differs depending on the polarization state of macrophages, and finally, whether MSCs of different origins respond differently to these signals. Our study revealed that macrophages constitute a powerful source of chemoattractants for MSCs, and the enhancement of both BM-MSCs and WJ-MSCs chemotaxis is highly significant (*p* < 0.01) regardless of the status of macrophages compared to the control. The more detailed analysis showed that the response of BM-MSCs and WJ-MSCs slightly differed between each other. WJ-MSCs responded significantly stronger to signals produced by M2 than M_IFNγ_ macrophages, whereas BM-MSCs migrated toward all analyzed subsets of macrophages to a similar extent. Finally, a direct comparison of the chemotactic abilities of BM-MSCs and WJ-MSCs showed that WJ-MSCs responded significantly stronger to chemotactic signals produced by M1 macrophages than BM-MSCs did. This difference might be related to the distinct expression of some chemokines receptors on BM-MSCs and WJ-MSCs, but this issue requires further investigation. Nevertheless, our results suggest that WJ-MSCs may migrate more effectively to tissues infiltrated with a high number of pro-inflammatory macrophages than BM-MSCs. We have additionally evaluated the mobility of MSCs under the influence of macrophages-derived secretomes with a scratch (“wound healing”) test. The assay showed that conditioned medium from unstimulated macrophages (CM-M0) significantly increased the scratch closure by WJ-MSCs (comparing to control, *p* < 0.05 in all time points), whereas the migration rate of BM-MSCs observed in parallel experiments remained unaffected. When we compared how the polarization state influences the secretome of macrophages in terms of its impact on MSCs mobility, we found that M_IFNγ_ and M1 macrophages significantly inhibited the mobility of both BM-MSCs and WJ-MSCs in comparison to M0 and M2 macrophages. It is possible that some factors secreted to a greater extent by the M2 subset than M1 macrophages stimulate the motility of MSCs. The potential candidates could be vascular endothelial growth factor C (VEGF-C) and transforming growth factor β (TGF-β), as they were relatively recently shown to promote the migration of mesenchymal stromal cells [42,43]. Alternatively, there are some soluble factors associated with M1 that inhibit the activity of the MSC in the scratch test. For example, Freytes et al. [14] showed that IL-1β (one of the typical cytokines secreted by M1) significantly reduces the viability of MSCs. Moreover, these authors showed that M1 macrophages and their associated cytokines, in general, inhibited hMSC growth, while M2 macrophages maintained or even enhanced hMSC growth, which is consistent with our data. On the other hand, Valles et al. [39], who, like us, analyzed the migration of MSCs under macrophage conditioned medium with the use of scratch assay, stated that CM from M1 macrophages enhance the migration of MSCs. Unfortunately, these authors present no numerical data from this experiment or any statistical analysis, and therefore it is difficult to relate our data to their conclusions.

The observation of differences between the analyzed cell types raises the question of what determines these differences. When MSCs from different sources are compared, the most obvious consideration for any differences between the various MSC types is the niche from which the cells are derived. For example, it is indicated that adipose-derived MSCs have a higher adipogenic potential than bone-derived MSCs. When comparing MSCs from adult organisms with WJ-MSCs, the question of donor age additionally arises. Since the WJ-MSCs are of fetal origin [44], the difference in the donor age seems to be, next to the niche, the primary, irremovable differentiating factor. Previous reports have indicated that the age of the donor may have an impact on the characteristics of the MSC, especially when the analysis is more detailed. Solving the problem of whether the differences between adult BM-MSCs and WJ-MSCs are more due to the age difference or more to the niche difference would require inclusion in the analysis of a third population: BM-MSCs from newborns. However, this is an unrealistic scenario for studies using human cells. On the other hand, it seems that resolving this issue is not crucial in terms of searching for optimal MSC candidates for specific clinical applications.

In summary, according to our knowledge, this is the first report that presents the direct comparison of human BM-MSCs and WJ-MSCs in terms of their interaction with macrophages. Our results indicate that both types of MSCs induce the polarization of macrophages toward the M2 phenotype to a similar extent. Moreover, both types of MSCs are strongly chemoattracted by macrophages in vitro. However, in addition to these important similarities, we also observed some differences—we showed that under pro-inflammatory stimulation, WJ-MSCs secrete significantly more IL-6 than BM-MSCs. Moreover, our data indicate that WJ-MSCs are significantly more responsive to M1-derived chemotactic signals than BM-MSCs. These results indicate that although both BM-MSCs and WJ-MSCs have the ability to reciprocally interact with macrophages, which may be the basis for immunomodulatory therapy, WJ-MSCs seem to be a slightly better candidate for future clinical settings in terms of immunomodulation.

## Data Availability

The datasets used and analyzed in the current study are available from the corresponding authors upon request.
